# Annotations

**Published:** 1901-08-24

**Authors:** 


### Annotations.

### Why do we Drink?

We now and again come across patients who assure us that they never drink, a statement which it is our first and perhaps most natural impulse to regard as an exaggeration. So indeed it may be, for on cross-examination such patients will probably say "Oh yes, I take a little soup of course." Still the general statement remains true that there are people who never drink in the sense that they never use cup or glass, and there certainly are a large number of people who drink only very little, ridiculously little compared with those around. These are healthy people going about their business calmly, doing good work, and often doing it with far greater comfort than those who, with glass in one hand and handkerchief in the other, mop their faces and declaim against the sweltering weather. Thus we are led to ask Why do we drink? and to answer that for the majority it is to a large extent a matter of habit and of self-indulgence. As the result of habit with many people, the slightest sense of thirst sets up longings which cannot be, or at least are not, resisted, with the result that much more fluid is taken than is wanted. "Are you very thirsty?" inquired the doctor. "Well no, sir, I takes good care o' that," replied the affable patient as he mopped his brow. Much of the constant drinking, not merely of alcoholics but of fluids of all kinds, to which one is tempted at every turn, is quite unnecessary and only leads to flabbiness and discomfort. In considering the amount of fluid we ought to drink we must always bear in mind the quantity of water which is contained in our ordinary food. According to Parkes we may take it that we require for ordinary work about three times as much water as of food (calculated dry), namely, about 75 per cent. of the whole intake, and if we look at the table in which he gives the amount of water contained in the various kinds of food, we find how many articles carry with them more than the required proportion. For instance, beef steak contains 74·4, white fish 78, poultry 74, potatoes 74, cabbages 91, carrots 85, vegetable marrows 95, and even dry bread contains 40 per cent. of water. As to fruits, apples contain 82 and strawberries 90 per cent. of water. Gravies, sauces, etc., and all forms of milk puddings, also contain more than the full average supply of water required, so that it evidently would be an easy task to arrange a diet which, although solid enough for all demands, would not require to be supplemented by actual drinking. If, however, we will but partake of fluid in moderation, say 40 ozs. or 50 ozs. a day, we are the better for it, for certain foods require the production of a not inconsiderable amount of digestive juices for their proper assimilation, and if we persist in eating so much meat as so many of us do a fair amount of water must be taken to wash out the nitrogenous waste which such a diet produces.

### Electricity in Treatment.

The discovery of the X-rays has undoubtedly attracted the attention of the medical profession towards electricity as a powerful therapeutic agent as well as a most useful ally in diagnosis. Many of the hospitals have now electrical departments, fitted with the latest appliances, and officered by special physicians, and we may therefore hope that in a few years a most valuable amount of data will have been collected. There is unfortunately a tendency to ride every new discovery to death, and we cannot expect electricity will cure baldness, skin diseases, and the thousand and one complaints which are now being treated by various forms and strengths of currents and light rays, but still there are many conditions which resist all drugs and are greatly benefited by electrical treatment. Nowadays, when everyone in the large towns has an electric main at his door, and most houses are lighted by electricity, nothing is easier than to fit up a transformer in the consulting-room, which can be used for cautery or for faradism at will. The old days of batteries which constantly required charging or cleaning and were never in order when they were particularly required have passed. Now it is merely necessary to switch on the current and everything is ready to hand. For some kinds of headache, with depression and brain fag, the benefit derived from a faradic current is instantaneous, and further is by no means fleeting in its character. More severe forms of neuroses and paralyses are also markedly benefited by currents appropriately given, either by means of pads or by baths. Unfortunately treatment by electricity has to a certain extent fallen into disrepute owing to the numerous unqualified people who habitually use it or invent and sell contrivances which either have no rational basis for their existence, or actually create no electricity at all. Again, an objection is often raised by physicians that they do not understand exactly the action of electricity; but this, surely, applies also to the majority of drugs which are prescribed on an empirical basis. It is therefore to be hoped that this will not delay practitioners from fitting up, wherever possible, appropriate transformers in their houses, for it is not impossible that with increased experience the therapeutic powers of electricity may prove to be far more useful than is generally believed.

### Moses and Tuberculous Meat.

It has been a fashion for some time back to regard Moses as the great sanitarian of antiquity, and on the discovery of anything new in hygiene to pick out from the Old Testament some text or other showing that there is nothing new under the sun, and that all that we are now preaching has been put in practice by the Jews from the earliest ages. Lately, we have heard much as to the examination to which all meat is submitted by the Jews, and it has been assumed by some that their rejection of carcases affected with tuberculosis is to be taken as showing that their code recognises the infective nature of this disease. Of course, it does nothing of the kind. As is well known, according to the Jewish law an examination of the viscera is made before a carcase is pronounced fit for food, and there are a considerable number of conditions, the discovery of which lead to its rejection. The reason of all this would appear to be to secure the rejection of the meat from any beast affected by any such disease as would have ultimately led to its death if the butcher had not intervened, and among these conditions are some which would of necessity involve the rejection of all advanced cases of tuberculosis. That is all. What may have been the original object of the Law giver we cannot now discuss, any more than we can the manner of the giving as described in biblical history. We may feel content, however, that there is nothing in either the law or the prophets to indicate any recognition of the pathological questions which are now exciting so much interest

